# Complications of Diabetes 2016

**DOI:** 10.1155/2016/6989453

**Published:** 2016-10-16

**Authors:** Konstantinos Papatheodorou, Nikolaos Papanas, Maciej Banach, Dimitrios Papazoglou, Michael Edmonds

**Affiliations:** ^1^Diabetes Clinic, Second Department of Internal Medicine, Democritus University of Thrace, 68100 Alexandroupolis, Greece; ^2^Department of Hypertension, Chair of Nephrology and Hypertension, Medical University of Lodz, Lodz, Poland; ^3^Diabetic Foot Clinic, King's College Hospital, London, UK

The prevalence of diabetes (DM) is constantly increasing worldwide at an alarming rate. According to the International Diabetes Federation in 2015, an estimated 415 million people globally were suffering from this condition [1]. Complications of DM account for increased morbidity, disability, and mortality and represent a threat for the economies of all countries, especially the developing ones [2]. The present special issue has been devoted to the recent progress in our understanding of diabetic complications, including the underlying molecular mechanisms, new diagnostic tools that facilitate early diagnosis, and novel treatment options. It consists of 20 articles covering 5 thematic areas: (a) epidemiology and pathogenesis of diabetic complications, (b) microvascular complications, (c) macrovascular complications, (d) miscellaneous complications, and (e) treatment options.

*(a) Epidemiology and Pathogenesis of Diabetic Complications*. There is growing evidence that the underlying mechanisms in the pathogenesis of diabetic complications include certain genetic and epigenetic modifications, nutritional factors, and sedentary lifestyle [3]. In a paper of this special issue entitled “Epigenetic Studies Point to DNA Replication/Repair Genes as a Basis for the Heritable Nature of Long Term Complications in Diabetes,” A. A. Leontovich et al., using a zebrafish diabetic model, have explored the role of epigenetic mechanisms on the persistence of diabetic complications even after euglycemic control is achieved, a condition known as metabolic memory. They found that DNA-methylation, in or near genes belonging to the DNA replication/DNA metabolism process group, might play a key role in this process. Regarding basic risk factors for macro- and microvascular complications, the Irish Longitudinal Study on Ageing (TILDA), as M. L. Tracey et al. describe in their article “Risk Factors for Macro- and Microvascular Complications among Older Adults with Diagnosed Type 2 Diabetes: Findings from The Irish Longitudinal Study on Ageing,” has recognized ageing, male gender, smoking, low level of physical activity, and high cholesterol as independent predictors of macrovascular complications. Conversely, smoking, hypertension, and duration of DM over 10 years proved to be predictive factors for microvascular complications.

*(b) Microvascular Complications.* Diabetic nephropathy, neuropathy, and retinopathy are the main microvascular complications induced by chronic hyperglycemia via several mechanisms such as the production of advanced glycation end products (AGEs), the creation of a proinflammatory microenvironment, and the induction of oxidative stress [4, 5].

Four articles in this special issue focus on diabetic nephropathy (DN). The first, by K. Sawada et al. entitled “Upregulation of *α*3*β*1-Integrin in Podocytes in Early-Stage Diabetic Nephropathy” shed light on the mechanism of podocyte detachment from the glomerular basement membrane, which is considered to be a key factor in the development of DN. The authors conclude that the early stages of this procedure are mediated by an upregulation of *α*3*β*1-integrin in podocytes. In the second article about DN entitled “Oxidative Stress in Diabetic Nephropathy with Early Chronic Kidney Disease,” A. G. Miranda-Díaz et al. have reviewed the effects of hyperglycemia-induced production of reactive oxygen species (ROS) on the renin-angiotensin system and the signaling pathway of the transforming growth factor-beta (TGF-*β*). They have concluded that oxidative stress leads to the production of chronic inflammation and the glomerular and tubular hypertrophy, which characterize the early stages of DN. Turning their attention to the diagnosis of early diabetic nephropathy, C. Gluhovschi et al., in another paper of this issue titled “Urinary Biomarkers in the Assessment of Early Diabetic Nephropathy,” have attempted to review novel biomarkers indicating renal injury such as transferrin, ceruloplasmin, podocalyxin, and VEGF. These markers can detect renal injury even before the presence of microalbuminuria, which still remains the most valid biomarker for DN in clinical practice. The last paper on the same topic, by X. Li et al., entitled “Histone Acetylation and its Modifiers in the Pathogenesis of Diabetic Nephropathy,” provides an overview of the potential involvement of epigenetic mechanisms, such as histone acetylation and other cellular processes in the development and progression of DN. It may be hoped that these mechanisms can help towards defining new therapeutic approaches for this microvascular complication of DM.

Three more articles have been included in the present special issue referring to diabetic retinopathy and neuropathy. The article entitled “Diabetic Retinopathy Is Strongly Predictive of Cardiovascular Autonomic Neuropathy in Type 2 Diabetes” has evaluated risk factors for cardiovascular autonomic neuropathy (CAN) in patients with T2DM. In this article, C.-C. Huang et al., using the deep breathing test, the Valsalva maneuver method, and the Composite Autonomic Scoring Scale to estimate the severity of autonomic neuropathy, found that diabetic retinopathy is the most significant predictive factor for CAN. In a further work, L. Forga et al. have conducted an observational, retrospective study in order to identify risk factors for the development of diabetic retinopathy (DR) in patients with type 1 DM. In their study entitled “Influence of Age at Diagnosis and Time-Dependent Risk Factors on the Development of Diabetic Retinopathy in Patients with Type 1 Diabetes,” they maintain that age at onset of type 1 DM, indexes of glycemic control, HDL-cholesterol levels, and diastolic blood pressure are all parameters predicting DR. In this regard, the study “Heart Rate Variability as Early Biomarker for the Evaluation of Diabetes Mellitus Progress” by R. E. Arroyo-Carmona et al. has used heart rate variability (HRV) as a tool to identify early diabetic complications and progress of DM in streptozotocin-induced diabetic mice.

*(c) Macrovascular Complications*. Atherosclerosis is more common in people with DM than in those without. For example, DM increases the risk for stroke in people aged 20 to 65 years more than 5 times [6]. The present special issue includes articles on macrovascular complications of DM as well. J. Zhang et al. in the article entitled “Coronary Plaque Characteristics Assessed by 256-Slice Coronary CT Angiography and Association with High-Sensitivity C-Reactive Protein in Symptomatic Patients with Type 2 Diabetes” have performed a coronary Computed Tomography Angiography to evaluate coronary plaque subtypes and luminal narrowing in patients with and without type 2 DM. They report that patients with DM are more prone to have significant stenosis with calcified plaques and such findings are accompanied by higher hs-CRP levels. Moreover, in a review article entitled “The Role of AGE/RAGE Signaling in Diabetes-Mediated Vascular Calcification,” A. M. Kay et al. emphasize the key role of AGE/RAGE signaling on the promotion of DM-mediated vascular calcification. In this process, many intracellular signaling pathways contribute to increased oxidative stress, which in turn leads to deposition of hydroxyapatite minerals into the extracellular matrix and vascular calcification. Furthermore, M. Samoš et al. in their work “The Impact of Type 2 Diabetes on the Efficacy of ADP Receptor Blockers in Patients with Acute ST Elevation Myocardial Infarction: A Pilot Prospective Study” have presented data from a prospective study that aimed to investigate platelet reactivity in patients with acute ST elevation myocardial infarction (STEMI) with or without T2DM, who have been treated with adenosine diphosphate (ADP) receptor blockers. Of note, this study has shown no difference between the two groups regarding platelet reactivity and the number of nonresponders to ADP receptor blockers. The last article of this thematic area is a retrospective quantitative study conducted in Australia. As B. T. Rodrigues et al. describe in their manuscript entitled “Prevalence and Risk Factors for Diabetic Lower Limb Amputation: A Clinic-Based Case Control Study,” ethnicity has been recognized as an independent risk factor for lower limb amputation in patients with diabetic foot, among whom indigenous Australians were most commonly affected.

*(d) Miscellaneous Complications*. Diabetic cardiomyopathy is a specific complication that develops independently of coronary artery disease or hypertension and it is possible to lead to increased morbidity and mortality [7]. The aim of the study “Assessment of Left Ventricular Structural Remodelling in Patients with Diabetic Cardiomyopathy by Cardiovascular Magnetic Resonance” by Y. Shang et al. was to evaluate the structural remodeling of left ventricular (LV) mass in patients with diabetic cardiomyopathy (DCM) using cardiovascular magnetic resonance (CMR). The authors contend that CMR can be a valid tool to estimate LV remodeling and its severity in patients with DCM. Y. Yu et al. in their article entitled* “*The Protective Effect of Low Dose Ethanol on Myocardial Fibrosis through Downregulating the JNK Signaling Pathway in Diabetic Rats” have explored the protective role of low dose ethanol on myocardial fibrosis in diabetic rats. In this study, low dose ethanol consumption was associated with lower mean arterial pressure, lower heart rate, high hydroxyproline content, and collagen volume fraction in myocardial tissue, together with decreased expression of ALDH2 and downregulation of the JNK pathway. Finally, in the review paper “Molecular and Electrophysiological Mechanisms Underlying Cardiac Arrhythmogenesis in Diabetes Mellitus,” G. Tse et al. discuss in detail the role of several cardiac factors (e.g., abnormalities in conduction or repolarization, electrophysiological, and structural remodeling) on arrhythmogenesis in patients with DM. They suggest that deeper investigation of these mechanisms can help towards defining new target molecules for potential future antiarrhythmic therapy for patients with DM.

*(e) Treatment Options*. The last thematic area covered by the present special issue relates to novel therapeutic options and it comprises four articles. In the first entitled “The Yin and Yang of the Opioid Growth Regulatory System: Focus on Diabetes: The Lorenz E. Zimmerman Tribute Lecture,” J. W. Sassani et al. provide an extensive overview of the role of the Opioid Growth Regulatory System on the development of diabetic complications. The authors have summarized all recent evidence indicating that certain pharmaceutical modifications in the function of this system can have profitable effects on diabetic animals. Clearly, there is a lot to learn about these intricate issues in the future. The second manuscript, “Implementation of a Diabetes Educator-Care Model to Reduce Paediatric Admission for Diabetic Ketoacidosis” by A. Deeb et al., has evaluated a diabetes educator-care model aiming to reduce the frequency of hospital admission of children and adolescents due to Diabetic Ketoacidosis (DKA). The authors have demonstrated that this model was an effective and sustainable measure for DM treatment achieving a significant reduction in the admission rate for DKA. L. Voroneanu et al. in their article entitled “Silymarin in Type 2 Diabetes Mellitus: A Systematic Review and Meta-Analysis of Randomized Controlled Trials” have conducted a comprehensive review and meta-analysis to evaluate the efficacy and safety of silymarin administration in patients with T2DM. They have found that this extract of milk thistle is an efficient and safe antidiabetic agent that might also have beneficial effects on renal function. However, significant heterogeneity and low quality of the available evidence were noted and lead to the need for further investigation of this issue. The final study pertains to the treatment of diabetic foot ulcers. M. Janka-Zires et al. in their article titled “Topical Administration of Pirfenidone Increases Healing of Chronic Diabetic Foot Ulcers: A Randomized Crossover Study” have conducted a randomized crossover study to assess the effect of topical administration of pirfenidone on noninfected chronic diabetic foot ulcers. Their findings confirm that the healing of these ulcers improves significantly by topical addition of pirfenidone.
